# Identification of a Novel Splice Site Mutation in *RUNX2* Gene in a Family with Rare Autosomal Dominant Cleidocranial Dysplasia

**DOI:** 10.52547/ibj.25.4.297

**Published:** 2020-10-19

**Authors:** Ebrahim Jamali, Raziyeh Khalesi, Fatemeh Bitarafan, Navid Almadani, Masoud Garshasbi

**Affiliations:** 1 Department of Genetics, School of Basic Science, Tonekabon Branch, Islamic Azad University, Tonekabon, Iran;; 2 Department of Medical Genetics, Faculty of Medical Sciences, Tarbiat Modares University, Tehran, Iran;; 3 Department of Cellular and Molecular Biology, North Tehran Branch, Islamic Azad University, Tehran, Iran;; 4 Department of Genetics, Reproductive Biomedicine Research Center, Royan Institute for Reproductive Biomedicine, ACECR, Tehran, Iran

**Keywords:** Cleidocranial dysplasia, RUNX2, Splice site

## Abstract

**Background:**

Pathogenic variants of *RUNX2*, a gene that encodes an osteoblast-specific transcription factor, have been shown as the cause of CCD, which is a rare hereditary skeletal and dental disorder with dominant mode of inheritance and a broad range of clinical variability. Due to the relative lack of clinical complications resulting in CCD, the medical diagnosis of this disorder is challenging, which leaves it underdiagnosed.

**Methods:**

In this study, nine healthy and affected members of an Iranian family were investigated. PCR and sequencing of all exons and exon-intron boundaries of *RUNX2 *(NM_001024630) gene was performed on proband. Co-segregation analysis was conducted in the other family members for the identified variant. Additionally, a cohort of 100 Iranian ethnicity-matched healthy controls was screened by ARMS-PCR method.

**Results:**

The novel splice site variant (c.860-2A>G), which was identified in the intron 6 of *RUNX2* gene, co-segregated with the disease in the family, and it was absent in healthy controls. Pathogenicity of this variant was determined by several software, including HSF, which predicts the formation or disruption of splice donor sites, splice acceptor sites, exonic splicing silencer sites, and exonic splicing enhancer sites. *In silico* analysis predicted this novel variant to be disease causing.

**Conclusion:**

The identified variant is predicted to have an effect on splicing, which leads to exon skipping and producing a truncated protein via introducing a premature stop codon.

## INTRODUCTION

Cleidocranial dysplasia/Dysostosis (CCD; MIM# 119600) or Marie and Sainton syndrome is a rare hereditary disease with autosomal dominant inheritance, clinically presented with congenital skeletal malformations of bones such as clavicular hypoplasia/aplasia, abnormal sutures and fontanelles, short stature, Wormian bones, and supernumerary teeth^[^^1^^]^. 

The first case of CCD was described by Martin with lack of clavicle^[^^2^^]^. In 1967, Keats^[^^3^^]^ reported additional manifestations for this disease, including abnormalities of long bones, the base of the skull, and the spine. Later, Jarvis and Keats^[^^4^^] ^comprehensively reviewed skeletal anomalies in CCD. 

CCD with an estimated prevalence rate of 1:1000,000 has been indicated to have complete penetrance^[^^5^^,^^6^^]^. Due to the relatively less medical complications in comparison with other skeletal dysplasia, CCD is most likely underdiagnosed. It may be seen at any age, and both sexes are affected approximately equally^[^^7^^]^. 

The mutations in *RUNX2* gene, located at chromosome 6p21, cause this disorder^[^^5^^,^^8^^]^. RUNX2 is a key transcription factor necessary for osteoblast differentiation. It binds to a DNA sequence element called RUNX-binding site (PyGPyGGT) and regulates number of bone-related genes^[^^9^^]^. Any mutation in *RUNX2* will cause defects in the membranous and endochondral bone formation^[^^5^^,^^6^^]^.

So far, few cases with CCD have been reported from Iran. Therefore, the mutation spectrum of Iranian CCD patients is still poorly understood. Herein, we report a novel mutation in *RUNX2* gene in an Iranian family with several affected members.

## MATERIALS AND METHODS

**Patient**

**Fig. 1:**
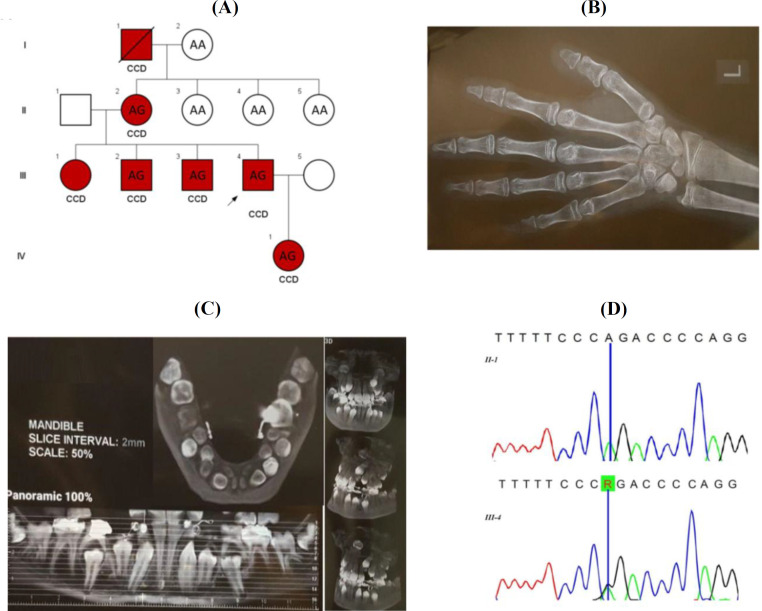
(A) The pedigree of the family with CCD and genotype of the studied samples for the c.860-2A>G variant in *RUNX2*. (B) Left hand X-ray of IV1 showing shortened middle phalange, hypoplastic distal phalanges, brachydactyly (especially the fifth digit), and mildly longed second metacarpus. (C) Panoramic mandibular X-ray of IV1 demonstrating dental caries, crowded, irregular and wide spaced teeth, and supernumerary teeth, occult, and unruptured multiple permanent teeth. (D) Sequencing chromatographs for the region of the c.860-2A>G variant in the *RUNX2* gene in one healthy (II-1) and one affected member (III-4) of the family. “R” designates purine (A or G) ribonucleotides

We studied nine healthy and affected members of a family in three generations (Fig. 1A). Affected individuals characterized by normal head circumference, mild frontal bossing, wide and prominent forehead, mild hypertelorism, broad and depressed nasal bridge, mild midface hypoplasia, crowded, irregular, and wide-spaced teeth, triangular face, no orofacial clefting, narrowed shoulder, short ribs, hypoplastic clavicles, narrowed chest, dental caries, shortened middle phalanges, hypoplastic distal phalanges, brachydactyly, mild lumbar lordosis, mildly longed second metacarpus, mild osteopenia, normal bone age, hypoplastic iliac wings and broad iliac notch (bilateral), multiple wormian bones in skull X-ray, supernumerary teeth, occult, and unruptured multiple permanent teeth, and proportionate short stature. Available skeletal radiography, including left hand and panoramic mandibular X-ray of IV1, are shown in Figure 1B and 1C, respectively. Additionally, a group of 100 healthy individuals from Iranian population consisting of 55 males and 45 females were randomly collected. The frequency of the identified novel variant was checked in this healthy control group by ARMS-PCR method.

**DNA extraction and primer design**

Approximately 5 mL of peripheral blood was collected from the proband, four other affected members, and four healthy individuals of this family. Genomic DNA was extracted using DNA isolation kit (Roche Life Science). Primers were designed in order to amplify all the coding regions and exon-intron boundaries of *RUNX2* gene (NM_001024630) using Primer3 software (Table 1).

**PCR sequencing**

Eight amplicons covering the exons and exon-intron boundaries of *RUNX2* were amplified using an initial denaturation step at 95 °C for 5 min, followed by 30 cycles with each at 95 °C for 40 s, annealing at the optimized temperatures, which are shown in Table 1, for 30 s, and 72 °C for 30 s, followed by final extension at 72 °C for 10 min. The PCR products were run on 2% agarose gel and were stained by ethidium bromide. All the PCR products were sequenced by an ABI 3730Xl sequencer machine. Sequencing results were analyzed by CodonCode Aligner software (v.7.0.1). 

**ARMS PCR**

PCR amplifications were performed in two distinct tubes. The total volume of each reaction was 25 μL and contained Taq DNA polymerase 2× master mix red, 2 mm of MgCl_2_ (Ampliqon, Denmark), primers at a final concentration of 0.2 pM, and 30 ng of genomic DNA. 

**Table 1:** Amplification primer sequences, PCR product size, and optimized annealing temperatures

	**Primer’s ID**	**Sequences**	**PCR ** **product (bp)**	**Optimized annealing temperatures (°C)**
**PCR-sequencing** **primers**	E1-RUNX2-F	TGCTCATTCTCTTTTTGTTTTG	598	61
	E1-RUNX2-R	TTTCTTCTGGTGAGGGTTAATG		
			
	E2-RUNX2-F	CCTGATAAGACACATCATTTGC	309	62
	E2-RUNX2-R	TCATCAAAGGAGCCTAATGTG		
			
	E3-RUNX2-F	CAGATGCTTCATTCCTGTCG	359	62
	E3-RUNX2-R	CATCAAAGGAGCCTAATGTGC		
			
	E4-RUNX2-F	TGCTGCTGTGTAATCATCAAC	230	62
	E4-RUNX2-R	CCTCATAGGGTCTCTGGAAAC		
			
	E5-RUNX2-F	TGGAAGGCATTATGTAGACAAG	397	62
	E5-RUNX2-R	ACATCTCCTCTGGTAGCCC		
			
	E6-RUNX2-F	AAGGCTGCAATGGTTGCTATAC	299	60
	E6-RUNX2-R	GTGAGCATGGATGAGACAGG		
			
	E7-RUNX2-F	TGCTTCTCCTTCTCTCTTGG	596	62
	E7-RUNX2-R	GCCCTTTTCTAATACCAGTGC		
			
	E8-RUNX2-F	TGGCTTGCTGTTCCTTTATG	653	64
	E8-RUNX2-R	TGATACGTGTGGGATGTGG		
				
**ARMS-PCR primers**	RUNX2-Fm	TACTAAAGATTTTTCTTTTTCTTTTTCACG	330	60
	RUNX2-Fn	TACTAAAGATTTTTCTTTTTCTTTTTCACA		
	RUNX2-RC	ATTTGCCAGTTGTCATTCCC		
			
	Internal control-F	TGCTCATTCTCTTTTTGTTTTG	598	
	Internal control-R	TTTCTTCTGGTGAGGGTTAATG		

The temperature protocol for PCR amplification was as follows: initial denaturation at 95°C for 5 min, followed by 30 amplification cycles of 30 s at 95°C, 30 s at 60°C and 30 s at 72°C, followed by final extension at 72°C for 10 min. The PCR products were run on 2% agarose gel and stained by ethidium bromide. Two samples with known genotypes were used as positive (heterozygote) and negative (homozygote for normal allele) controls.

***In silico*** **prediction of pathogenicity**

In this study, pathogenicity of the discovered variant was determined by several software such as Mutation tasting (http://www.mutationtaster.org/), VEP (http:// www.ensembl.org/Tools/VEP) and HSF (http://www. umd.be/HSF/). The frequency of this variant was checked in 1000 genome project, ExAC, and Iranome databases.

**Ethical statement**

The above-mentioned sampling protocols were approved by the Research Ethics Committee of Islamic Azad University, Tonekabon Branch, Tonekabon, Iran (Ethical code: IR.IAU.TON.REC.1399.031). Written informed consent was provided by all the participants in the study.

## RESULTS

A novel splice site variant (c.860-2A>G) in the intron 6 of *RUNX2 *gene was identified in the proband by Sanger sequencing. This variant probably will result in a premature stop codon and truncated protein. The variant was observed in the other affected members but was absent in the healthy individuals (Fig. 1D). We could not observe this variant in 100 healthy ethnicity-matched control individuals by ARMS-PCR. The variant was also absent in 1000 genome project, ExAC, and Iranome databases. Different online *in silico* prediction software such as MutationTasting, HSF, and VEP indicated that this variant is pathogenic. 

## DISCUSSION

To date, four types of CCD have been reported. The first type has typical clinical manifestations and is hereditary, the second one has typical clinical manifestations but is not hereditary, the third group has atypical clinical manifestations and is hereditary, and the fourth type has neither typical clinical manifestations nor a history of heredity^[^^10^^]^. Herein, we observed typical clinical manifestations of CCD and history of heredity through four generations in an Iranian family. 

One of the prominent clinical findings in affected individuals with CCD is clavicular hypoplasia, which has also been reported in several other cytogenetic abnormalities^[^^5^^]^. Translocations and duplications including 8q22^[^^11^^]^, partial trisomy 11q^[^^12^^]^, partial trisomy 11q/22q^[^^13^^]^, trisomy 20p^[^^14^^]^, and submicroscopic deletions occur in a small fraction of CCD patients^[^^15^^,^^16^^]^. In this study, no cytogenetic abnormality was found in affected individuals.

While there is a wide range of clinical manifestations in CCD patients^[^^10^^]^, many other diseases resemble CCD^[^^17^^]^; for instance, Pyknodysostosis (MIM 265800), a rare osteosclerosis disease characterized by malformations of sutures and fontanelles, Wormian bones, clavicular hypoplasia, short terminal phalanges, and tooth anomalies^[^^5^^]^. The major clinical manifestations of mandibuloacral dysplasia (MIM 248370) are clavicular hypoplasia, Wormian bones, persistent cranial sutures, and dental problems. Patients suffering from Yunis-Varon syndrome (MIM 216340) also have clavicular hypoplasia. 

The RUNX proteins are key regulators of bone formation^[^^17^^]^. CCD is caused by haploinsufficiency of the RUNX2 transcription factor^[^^16^^,^^18^^]^. Mutations in *RUNX2* gene have high penetrance and extreme variability, ranging from isolated dental anomalies to fully manifesting disease with poorly ossified cranium and absence of clavicles^[^^10^^]^. The significance of RUNX2 is highlighted as a key regulatory factor in bone formation by studies on mice models. Mice carrying one disrupted *RUNX2* allele resembled the human CCD phenotype, including clavicular hypoplasia, and the homozygous Runx2 mutant mice revealed disturbing bone formation due to the absence of ossification^[^^17^^]^. Until now, over 100 different mutations^[^^19^^]^ have been reported in the *RUNX2* gene^[^^20^^-^^22^^]^, including missense, nonsense, splicing, and frame-shift variants in over 65% of CCD cases^[^^21^^-^^23^^]^. In this study, a novel splice site mutation of c.860-2A>G in the *RUNX2* gene was identified. *RUNX2* contains several domains: (i) an N-terminal Q/A domain composed of a stretch of 23 consecutive glutamines, followed by 17 consecutive alanines, which the length of this domain is important for transcriptional activity^[^^24^^-^^26^^]^; (ii) a RUNT domain, that has a unique ability of mediating DNA binding and protein heterodimerization; (iii) a C-terminal proline-serine-threonine-rich activation domain that can also mediate protein-protein interaction^[^^24^^]^ and it is essential for the transcriptional activity of RUNX2 protein^[^^23^^]^; (iv) the last five amino acids of the protein known as the VWPRY motif^[^^19^^]^. 

The novel splice site variant, c.860-2A>G, in the *RUNX2* gene probably results in a frameshift in the reading frame after proline-serine-threonine-rich domain via exon skipping and eventually causes a premature stop codon. The intact VWRPY motif has been proposed to be crucial for the transcriptional repression activity of RUNX2 protein via interaction with TLE proteins, which is essential for osteogenesis^[^^19^^]^. Therefore, we can conclude that the c.860-2A>G splice site variant identified in our study will likely remove the VWRPY peptide sequence part of RUNX2 due to the premature stop codon.

 CCD disorder shows a broad spectrum of phenotypic variability ranging from cases who do not exhibit typical symptoms to classic clinical manifestations^[^^27^^]^. In the current study, the classical manifestations of CCD patients have been detected in an Iranian family. Furthermore, a splice site mutation (c.860-2A>G) was found in the intron 6 of *RUNX2* gene in this family, which has not been reported before. Overall, clinical diagnosis can play a key role in identifying CCD patients and recommends that Sanger sequencing of *RUNX2* gene can be a rapid and cost-efficient diagnostic solution for CCD families who show classical manifestations of this disease.
